# 1503. Impact of Sars-Cov-2 (Covid-19) in Patients Living with HIV from United States National Inpatient Sample Database

**DOI:** 10.1093/ofid/ofad500.1338

**Published:** 2023-11-27

**Authors:** Premalkumar M Patel, Daniel Acosta, Esha Vallabhaneni, Fernando Poli De Frias, Yavani Singh, Christopher Lopez, Claudio Tuda

**Affiliations:** Mount Sinai Medical Center of Florida, Hollywood, Florida; Mount Sinai Medical Center, Miami Beach, Florida; Mount Sinai Medical Center, Miami Beach, Florida; Mount Sinai Medical Center, Miami Beach, Florida; Mount Sinai Medical Center, Miami Beach, Florida; Mount Sinai Medical Center, Miami Beach, Florida; Mount Sinai Medical Center, Miami Beach, Florida

## Abstract

**Background:**

The COVID-19 pandemic has raised concerns about the impact on those living with HIV/AIDS due to weakened immune systems. Disruptions to HIV services and medication supplies caused by the pandemic have also raised concerns about increased HIV-related illnesses and deaths. An inpatient sample database was used to investigate the impact of Covid-19 on HIV patients in the US by examining the clinical outcomes of Covid-19 patients with and without HIV in US hospitals.

**Methods:**

The NIS database is a publicly available and extensive database that contains information on millions of hospitalizations in the United States. Using the NIS 2020 database, adult patients who were diagnosed with COVID-19 were identified and categorized based on their HIV status. In order to examine the NIS database, a variety of statistical approaches were employed, such as univariate and multivariate analysis, descriptive statistics, and regression analysis.

**Results:**

A total of 11,080 hospitalized HIV-positive patients were diagnosed with COVID-19. Amongst them, 7,510 were male and 3,570 were female with 23.1% being Caucasian, 50.8% African American, and 19.3% Hispanic. 47% of the patients had a national median household income between 0-25th percentile. Geographically, 27.8% were from the northeast, 19.4% from the midwest, 44.4% from the south, and 8.4% from the west. The most common comorbidities were hypertension at 32.8%, diabetes mellitus with complications at 27.8%, chronic lung disease at 26.7%, obesity at 22.2%, hypothyroidism at 7.6%, drug abuse at 8.6%, and depression at 14.5%. The mean age for patients with HIV who contracted COVID-19 was 56 years with a length of stay (LOS) of 9.7 days and an economic burden of $27,566 in comparison to patients without HIV, who had a mean age of 62 years, LOS of 7.9 days, and cost of $21,553.

Data Analysis : Demographics

**Figure:**
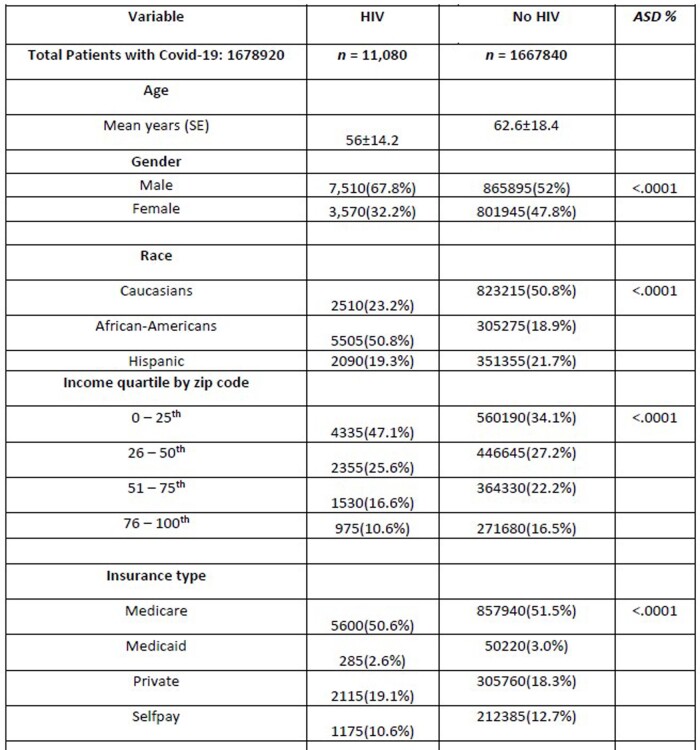

Data Analysis: Comorbidities

**Figure:**
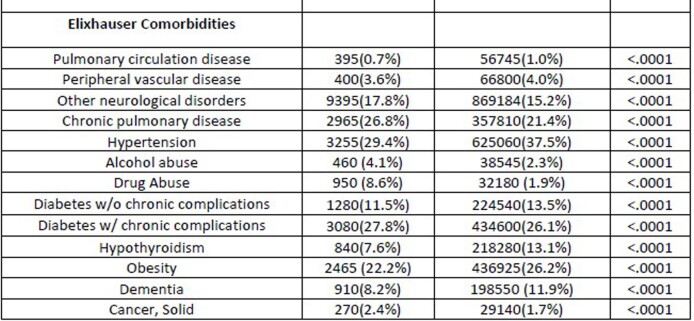

Data Analysis: Outcomes

**Figure:**
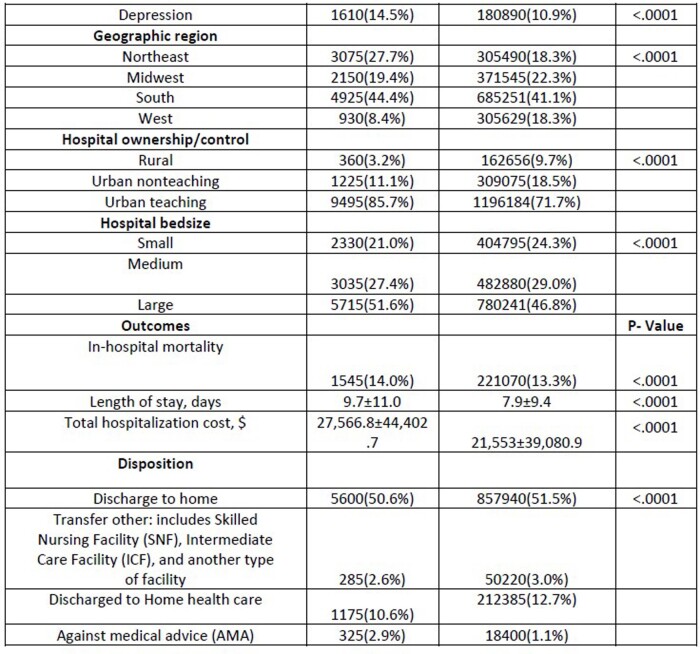

**Conclusion:**

In conclusion, hospitalized patients living with HIV, particularly African Americans, face a greater risk for adverse outcomes from COVID-19, which results in increased healthcare utilization. This underscores the need for safeguarding vulnerable populations and monitoring infectious disease spread. Further research is needed to better understand how COVID-19 affects different demographics and how to address their healthcare needs during pandemics.

**Disclosures:**

**All Authors**: No reported disclosures

